# 

**Published:** 1842-01

**Authors:** 


### BRITISH AND FOREIGN

### MEDICAL REVIEW

OR

### QUARTERLY JOURNAL

OF

### PRACTICAL MEDICINE AND SURGERY

STATE OF LOUISIANA CHARITY HOSPITAL
JUSTICE
MEDICAL LIBRARY

LIBRARY OF THE

Orleans Parish Medical Society

EDITED BY

JOHN FORBES M.D. F.R.S. F.G.S.

---

VOL. XIII.

JANUARY—APRIL 1842.

LONDON
JOHN CHURCHILL PRINCES STREET SOHO.

MDCCCXLII.

LONDON:

PRINTED BY C. ADLARD, BARTHOLOMEW CLOSE.

### BOOKS RECEIVED FOR REVIEW.

ENGLISH.

1. The Oily Acids, forming the first Supplement to the Seventh Edition of Dr. Turner's Chemistry. 4s.

2. A Practical Treatise on the New Operation for Lateral Curvature of the Spine. With plates. By G. B. Childs, M.R.C.S. —London, 1841. 8vo, pp. 56. 4s.

3. A Concise and Practical Treatise on the principal Diseases of the Air-passages, Lungs, and Pleura. By A. Catherwood, M.D. C.M. (?)—Lond. 1841. 8vo, pp. 208. 7s. 6d.

4. Pathology founded on the Natural System of Anatomy and Physiology; a Philosophical Sketch. By Alexander Walker.—Lond. 1841. 8vo, pp. 162. 5s. 6d.

5. Oratio in theatro Collegii regii Medicorum Londinensis, in Harveii instituto habitu die. Julii xxv., An. M.DCCC.XLI. A Thoma. Mayo, M.D. Lon. 1841. 4to, pp. 16.

6. Guy's Hospital Reports. No. XXX. Oct. 1841. 6s.

7. Clinical Researches on Auscultation of the Respiratory Organs, and on the First Stage of Phthisis. By J. Fournet. Translated from the French by T. Brady, M.B. —London. 8vo, pp. 227. 7s.

8. The Diseases of Children, their Symptoms and Treatment; a Treatise intended for the use of the Student and Junior Practitioner. By G. A. Rees, M.R.C.S.—Lond., 1841. 8vo, pp. 300. 5s.

9. A Treatise on the Nature, Causes, and Treatment of Erysipelas. By T. Nunneley, Lecturer of Anatomy at the Leeds School of Medicine.—London, 1841. 8vo, pp. 307. 10s. 6d.

10. Principles of General and Comparative Physiology, intended as an introduction to Human Physiology, &c. By W. B. Carpenter, M.D., Lecturer on Physiology at the Bristol Medical School. Second Edition.—London, 1841. 8vo, pp. 577. 18s.

11. Illustrations of the Nervous System. By Joseph Swan. Part VII. and last.—London, 1841. 10s. 6d.

12. The Anatomy of the Arteries. By Richard Quain, Prof. of Anatomy in University College. Part IX.—Lon. 1841. 12s.

13. Researches into the Causes, Nature, and Treatment of the more prevalent Diseases of India, and of warm climates generally. By James Annesley, F.R.S. F.S.A. 2d Edit.—Lond., 1841. 8vo, pp. 606. 12s.

14. On the Remote Causes of Epidemic Diseases. By John Parkin, M.D., &c.—London, 1841. 8vo, pp. 198.

15. Treatise on the Oleum Jecoris Aselli, or Cod Liver Oil, as a therapeutic agent in certain forms of Gout, Rheumatism, and Scrofula; with Cases. By J. H. Bennett, M.D., &c.—London, 1841. 8vo, pp. 180. 6s.

16. Observations on the Analogy between Ophthalmic and other Diseases: being the substance of a paper read at King's College, London. By Charles Vines, M.R.C.S., Surgeon to the Reading Dispensary.—London, 1841. 8vo, pp. 32.

17. Surgery. From the Seventh Edition of the Encyclopædia Britannica. 4to, pp. 26.

18. Six Letters addressed to the President of Bethlem Hospital, respecting the Management of that Establishment. By Philanthropos.—London, 1841. 8vo, pp. 30. 1s.

19. Outlines of Comparative Anatomy. By R. E. Grant, M.D. F.R.S., &c.—London, 1841. 8vo, pp. 656. 28s.

20. On the present State of the Medical Profession in England; being the Annual Oration delivered before the Members of the British Medical Association. 21st Oct. 1841. By R. E. Grant, M.D. F.R.S. L. and E. —London, 1841. 8vo, pp. 98. 2s. 6d.

21. A Manual of General Therapeutics; with Rules for Prescribing, and a copious Collection of Formulæ. By D. Spillan, M.D. A.M.—Lond. 1841. 8vo, pp. 458. 10s. 6d.

22. An Examination of Reviews contained in the British and Foreign Medical Review, and the Medico-Chirurgical Review, of the Medical and Physiological Commentaries. By the author, Martin Payne, M.D.—New York, 1841. 8vo, pp. 96.

23. Elements of Chemistry. By R. Kane, M.D. M.R.I.A.—Dublin, 1841. 8vo, pp. 1204. 24s.

24. The Influence of Tropical Climates on European Constitutions. By James Johnson, M.D., and J. R. Martin, Esq. Sixth Edition, revised and greatly improved. —London, 1841. 8vo, pp. 693. 18s.

25. Pilgrimages to the Spas in Pursuit of Health and Recreation; with an Inquiry into the Comparative Merits of different Mineral Waters, the Maladies to which they are applicable, and those in which they are injurious. By James Johnson, M.D.—London, 1841. 8vo, pp. 299. 9s.

26. Elements of Physiology. By J. Müller, M.D., &c. Translated from the German, with Notes, by William Baly, M.D. With plates. (Complete.)—London, 1842. 2 vols. 8vo, pp. 1715. 2*l*.

27. Elements of the General and Morbid Anatomy of Man and the Mammalia. By F. Gerber, Prosector of the Universities of Bern. With Notes and an Appendix by George Gulliver, F.R.S.—Lond. 1842. 8vo, pp. 390 and 106. With a vol. of plates. 24s.

28. On the Employment of the Microscope in Medical Studies. A Lecture introductory to a course of Histiology. By J. H. Bennett, M.D.—Edin., 1841. 8vo, pp. 27.

